# Effect of Antenatal Parasitic Infections on Anti-vaccine IgG Levels in Children: A Prospective Birth Cohort Study in Kenya

**DOI:** 10.1371/journal.pntd.0003466

**Published:** 2015-01-15

**Authors:** Indu Malhotra, Maxim McKibben, Peter Mungai, Elisabeth McKibben, Xuelei Wang, Laura J. Sutherland, Eric M. Muchiri, Charles H. King, Christopher L. King, A. Desiree LaBeaud

**Affiliations:** 1 Case Western Reserve University, Center for Global Health and Diseases, Cleveland, Ohio, United States of America; 2 Case Western Reserve University, Clinical and Translational Science Collaborative, Cleveland, Ohio, United States of America; 3 Children’s Hospital Oakland Research Institute, Center for Immunobiology and Vaccine Development, Oakland, California, United States of America; 4 Division of Vector Borne and Neglected Tropical Diseases, Ministry of Public Health and Sanitation, Nairobi, Kenya; Universidad Peruana Cayetano Heredia, PERU

## Abstract

**Background:**

Parasitic infections are prevalent among pregnant women in sub-Saharan Africa. We investigated whether prenatal exposure to malaria and/or helminths affects the pattern of infant immune responses to standard vaccinations against *Haemophilus influenzae* (Hib), diphtheria (DT), hepatitis B (Hep B) and tetanus toxoid (TT).

**Methods and Findings:**

450 Kenyan women were tested for malaria, schistosomiasis, lymphatic filariasis (LF), and intestinal helminths during pregnancy. After three standard vaccinations at 6, 10 and 14 weeks, their newborns were followed biannually to age 36 months and tested for absolute levels of IgG against Hib, DT, Hep B, and TT at each time point. Newborns’ cord blood (CB) lymphocyte responses to malaria blood-stage antigens, soluble *Schistosoma haematobium* worm antigen (SWAP), and filaria antigen (BMA) were also assessed. Three immunophenotype categories were compared: i) tolerant (those having *Plasmodium*-, *Schistosoma*-, or *Wuchereria*-infected mothers but lacking respective Th1/Th2-type recall responses at birth to malaria antigens, SWAP, or BMA); ii) *sensitized* (those with infected/uninfected mothers *and* detectable Th1/Th2-type CB recall response to respective parasite antigen); or iii) unexposed (no evidence of maternal infection or CB recall response).

Overall, 78.9% of mothers were infected with LF (44.7%), schistosomiasis (32.4%), malaria (27.6%) or hookworm (33.8%). Antenatal maternal malaria, LF, and hookworm were independently associated with significantly lower Hib-specific IgG. Presence of multiple maternal infections was associated with lower infant IgG levels against Hib and DT antigens post-vaccination. Post-vaccination IgG levels were also significantly associated with immunophenotype: malaria-tolerized infants had reduced response to DT, whereas filaria-tolerized infants showed reduced response to Hib.

**Conclusions:**

There is an impaired ability to develop IgG antibody responses to key protective antigens of Hib and diphtheria in infants of mothers infected with malaria and/or helminths during pregnancy. These findings highlight the importance of control and prevention of parasitic infections among pregnant women.

## Author Summary

Parasitic infections are prevalent among pregnant women in sub-Saharan Africa. Prenatal exposure to parasitic infections can generate several potential effects on fetal immune responses and affect functional antibody generation during subsequent vaccination. There is a paucity of data on the detrimental effects of chronic parasitic infections during pregnancy on the response to vaccine from birth to childhood. This paper highlights the overwhelming presence of helminth infection and malaria in pregnant women in rural Kenya. The study shows that the presence of single and multiple antenatal parasitic infections is associated with impaired infant IgG levels against *Haemophilus influenzae* (Hib) and diphtheria (DT) antigens post-vaccination from birth to 30 months of age. This study found that the response to DT was reduced in malaria-tolerized infants, and the response to Hib was impaired in filarial-tolerized infants; by contrast, the *Schistosoma*-tolerized group showed no effect. Deworming campaigns must be directed towards pregnant mothers, infants, and young children to improve response to vaccination.

## Introduction

Vaccine-preventable diseases (VPD) continue to kill an estimated one to two million children each year, comprising about 14% of global mortality in children under 5 years of age [1, 2]. Vaccination studies have repeatedly shown that children in developing nations are less responsive to vaccines than children from developed countries [3–7]. Recent outbreaks of polio in Africa and Syria have also shown that vaccine failures can result in resurgence of vaccine preventable diseases even in countries with high vaccination coverage [8, 9]. This suggests that vaccine efficacy can be lower in certain settings. That is, failure to respond appropriately to vaccination is likely to be associated with poverty-related conditions, including malnutrition and chronic infection, particularly chronic parasitic infections [10, 11]. Animal models suggest that nematode infections decrease vaccine efficacy and contribute to risk for infection by vaccine preventable diseases [12, 13]. Concurrent schistosomiasis infection in humans has a negative impact on vaccination for tetanus [14] and tuberculosis (BCG vaccination) [15]. Chronic parasitic infections other than helminths can impair immune responses, resulting in decreased tetanus, *Haemophilus influenzae* type b (Hib), and typhoid vaccine efficacy in the presence of malaria infection [16, 17].

The immune consequences of parasitic infections can be reflected in the unborn children of infected mothers. Prenatal exposure to parasitic infections can generate a number of effects on fetal immune responses, and can affect functional response to post-partum vaccination, as we and others have shown for BCG [18–21]. Over the past decade, we have studied the influence of chronic maternal parasitic infections (lymphatic filariasis, schistosomiasis and malaria) on immune response in newborns and young children living in Kenya [18–20, 22–25]. It appears that transplacental trafficking of parasite antigens from mother to fetus occurs on a frequent basis, leading to multivalent T and B cell responses to parasitic infections in the newborn [20, 26–31]. This fetal priming results in two phenotypes: those that have an enhanced response to the parasite antigen (“*sensitized*”, with cord blood lymphocytes (CBL) producing IFNγ, IL-2, IL-4, and IL-13 to parasite antigen challenge) and those that have a suppressed response (“*tolerized*”, where CBL do not produce parasite antigen-induced IFNγ, IL-2, IL-4 or IL-13, but do produce IL-10 to parasite antigen challenge) [23, 32]. The goal of the present study is to determine how the individual and combined antenatal parasitic infections, and the resulting sensitization or tolerization of infant immune responses, could influence early childhood responses to standard *H. Influenzae* type B, diphtheria toxoid, tetanus toxoid, and hepatitis B virus vaccination.

## Methods

### Ethics statement

Approval for the study was obtained from the Kenya Medical Research Institute National Ethical Review Committee and from the Institutional Review Board for Human Studies at University Hospitals of Cleveland Case Medical Center. Mothers provided written informed consent for their own participation and that of their infants.

### Study design and study participants

Healthy pregnant women and their offspring born at the Msambweni District Hospital on the south coast of Kenya were enrolled in this mother-child cohort study. Mothers underwent a detailed questionnaire that queried their education level, spouse’s occupation, and household income. Women enrolled in the study were given malaria prophylaxis consisting of two single doses of sulfadoxine–pyrimethamine (SP) at the beginning of the second and third trimester, respectively, of pregnancy, and a single dose of albendazole (400mg) in accordance with recommendations from the Kenya Ministry of Health. Mothers and children were also examined and tested for parasitic infections at times of any intercurrent acute illnesses during the follow-up period, and treated appropriately.

Pregnant women provided venous blood, urine, and stool at their first antenatal clinic visit and again at delivery. For the mother-infant pairs, maternal venous blood, placental intervillous blood, and umbilical cord blood were collected at delivery, as previously described [18]. Infant venous blood, urine and stool samples were collected beginning at 6 mo. of age and every 6 mo. thereafter until age 36 mo. Plasma was stored at -80ºC until antibody assays were performed. Cellular immune response at birth was performed on fresh cells. Infants received standardized immunizations provided by the Ministry of Health following established Kenya National Health Service guidelines. Pentavalent (diphtheria-tetanus-whole cell pertussis-hepatitis B-Hib) vaccine was given at 6, 10, and 14 weeks, oral trivalent polio was given at birth, 6, 10, and 14 weeks and one dose of measles vaccine was given at 9 months. At birth, and at each 6-month follow up visit, length/height, weight, and head circumference were measured.

### Maternal and infant infection status

Maternal venous blood, intervillous placental blood, cord blood, and infant venous blood were examined for malaria infection status by light microscopy. DNA was extracted from blood and tested for *Plasmodium falciparum* (Pf) by real time quantitative PCR (RTQPCR) [33]. A newborn was considered “exposed” to malaria *in utero* if one or more of the blood smear preparations or RTQPCR results were positive from antenatal, placental, or cord blood testing. A newborn was considered “not exposed” when both diagnostic tests were negative on all specimens. Stool and urine were also obtained from infants at every 6 month visit and examined for the presence of intestinal helminths and *S. haematobium* ova as described previously [19, 20]. Infection status with *S. haematobium* was also assessed by ELISA detection of SWAP-specific (soluble worm antigen of *S. haematobium*) IgG4 antibodies in plasma samples collected [19, 20]. *Wuchereria bancrofti* infection was detected by assay for circulating microfilaria antigen in plasma samples with the use of a commercial Og4C3 antigen detection assay (TropBioMed, Townsville, Australia) and also assessed by ELISA detection of BMA-specific (*Brugia malayi* antigen) IgG4 antibodies [19, 20].

### Cord blood lymphocyte cultures

CBMC were isolated from fresh cord blood and were cultured in the presence of malaria antigens for recall responses to malaria as described [18]. For schistosomiasis, SWAP was used as the antigen challenge, and for LF, *Brugia malayi* antigen, (BMA), was used as the antigen marker for anti-filarial immune response, as this antigen is preserved across species and can be used to detect exposure to *W. bancrofti*, the filarial worm endemic to Kenya [19, 20]. All culture supernatants were collected at 72 h and immediately frozen at -80ºC for storage, pending cytokine assays. Quantification of IFNγ, IL-5 and IL-13 was performed on culture supernatants by ELISA and positive response was scored as previously described [18].

### Measurement of plasma IgG levels in response to Hib, DT, HepB and TT vaccinations

Response to vaccination was determined by standard ELISAs for IgG levels against tetanus toxoid (TT), diphtheria toxoid (DT), Hepatitis B virus (Hep B), and Hib (using its polyribitol phosphate (PRP) antigen) [34–36]. Briefly, ELISA plates were coated with 1 µg/ml of TT (Massachusetts Biolabs, Cambridge, MA); 0·5 LF/ml (flocculation unit/ml) of DT (diphtheria Antitoxin Human Serum NIBSC code: 00/496); 1µg/ml of Hep B surface antigen (*adw* subtype, Fitzgerald Industries, Concord, MA, USA) or 1 µg/ml *H. influenza* type b oligosaccharide-human serum albumin conjugate (ATCC: NR-12268) and incubated overnight at 4ºC. After blocking and washing, diluted plasma samples were added to the plates and incubated for 1 h at room temperature. The plates were washed and alkaline phosphatase-conjugated anti-human IgG (Jackson ImmunoResearch, Malvern, PA) was added at 1:1000 for 1 h at 37°C. Substrate orthophenoylenediamine (o-p-NN) was added after the final wash. The reaction was stopped by adding 5% EDTA, and absorbance was read at 405 nm with an ELISA reader. For all assays, standard sera obtained from NIBSC (National Institute of Biological Standards and Control) were used to create standard curves for determination of the subjects’ anti-antigen IgG concentrations.

### Effects of sensitization and tolerization on response to vaccine antigens

To assess potential mechanisms of altered vaccine responses in some offspring of women with parasite infection, we hypothesized that prenatal exposure to one or more parasite antigens would generate a tolerogenic immunophenotype, such that upon exposure to vaccines in early childhood, a bystander effect might alter the ability to generate a robust immune response. Children were classified as either sensitized, tolerized, or unexposed to the individual parasite pathogens studied.

This classification was based on their mother’s malaria, *Schistosoma*, and lymphatic filarial infection status and on their detectable Th1/Th2-type cord blood (CB) recall responses to each of these respective parasite’s antigens. The tolerant classes were defined as: i) *tolerant to malaria* if children had mothers with malaria (*P. falciparum*-positive by blood smear and/or PCR) but lacked detectable Th1/Th2-type CB recall responses to malaria blood stage antigens; ii) *tolerant to Schistosoma* if mothers had schistosomiasis, (SWAP IgG4 and/or *S. haematobium*egg positive) but lacked detectable Th1/Th2-type CB recall responses to SWAP antigens, and iii) *tolerant to LF* if mothers had evidence of LF, (BMA IgG4 and/or Og4C3 antigen positive) but lacked detectable Th1/Th2-type CB recall responses to BMA antigens. Sensitized infants were defined as *sensitized to malaria* if cord blood lymphocytes had detectable Th1/Th2-type recall responses to malaria, *sensitized to Schistosoma* if there was a SWAP-specific recall response in CB, and *sensitized to LF* if there was a BMA-specific recall response in CB; the mothers may or may not have been tested positive for the respective infection during delivery. SWAP and BMA were used at 25 µg/ml and 10 µg/ml respectively; these concentrations of antigens failed to induce an immune response in North American controls. *Unexposed* classes were defined as children who had mothers *P. falciparum*-negative, anti-SWAP-negative, and who also had a lack of CB lymphocyte responses individually to malaria, *Schistosoma* or LF antigens.

### Statistical analysis

Demographic characteristics of participating mothers were summarized using descriptive statistics. Pearson chi-squared test was used to compare maternal infection rates among different demographic groups, including age, education, household income, ethnic group and parity. To investigate the association between maternal infections and infants’ IgG responses to DT, Hep B, Hib and TT during the first 30 months of life, mixed-effects model was used to model infants’ IgG responses over time. A random intercept and slope were included to account for between-subject variance in vaccine response over time. A quadratic function of age time was also included as a fixed effect accounting for the natural curvature of infant vaccine response during infancy and early childhood. To avoid over-fitting models, covariates were selected *a priori* based on biological knowledge and included characteristics determined at enrollment: maternal age, parity, sex of infant, education and income level of the mothers, and maternal dose of tetanus (for IgG response to TT only), and characteristics collected during the study (infant parasitic infections at their follow-up visits). Due to very low infant infection rates of schistosomiasis, LF and hookworm, only infant malaria infection was included in the model when studying maternal malaria infections. Similar models were used to investigate the effect of sensitization and tolerization on infants’ IgG responses to DT, Hep B, Hib and TT during the first 30 months of life. SAS Version 9.2 was used for all analyses. Statistically significant differences were assessed using P < 0∙05 as criterion.

## Results

### Study population

**Figure 1:**
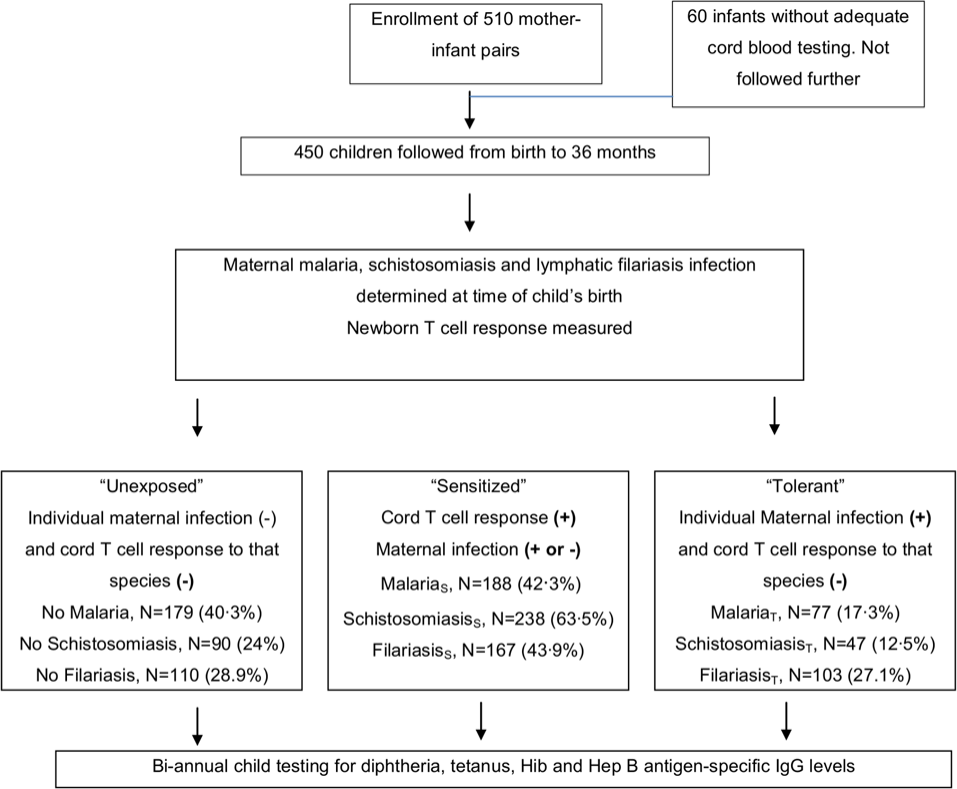
Flow diagram of birth cohort study.

An overview of the enrollment and follow up of participants is presented in Fig. 1. A total of 510 mother-newborn pairs were recruited between 2006 and 2009; of those, 450 children had informative cord blood testing and returned at least once after delivery, and were included in the analysis. For assessment of post-vaccination antibody responses, study children were followed every 6 months up to 36 months of age. The mean follow up time was 27 months (SD = 2∙7 months). Children were followed on an average of three to four times. Children were retained in the study if they missed one or more follow-ups, 299, 294, 246, 254 and 138 infants were followed at 6, 12, 18, 24 and 30 months respectively. The number of children followed at 36 months of age was small. Therefore, the 36 month outcomes were not included in the analysis. The primary reason for loss to follow-up was permanent emigration from the study area. There was no difference in the average number of maternal infections, age, or parity between the group of mother-infant pairs who dropped out and those who remained in the study.

### Characteristics of mothers

The demographic characteristics of the participating mothers, along with their rates of malaria, schistosomiasis, lymphatic filariasis and soil-transmitted-helminth infections are shown in Table 1. Mothers’ age showed a significant effect on the prevalence of malaria, *S. haematobium*, and soil-transmitted helminths (STH). The level of maternal education was inversely related to rates of STH (P<0.0001). Mothers from low-income households had more schistosomiasis than mothers from high-income households (P = 0∙003). There was no significant difference in infection rates according to mother’s gravidity or ethnicity.

**Table 1:** Characteristics of pregnant women enrolled in the study 2006–2009.

**Characteristic**		**Malaria**		**Schistosomiasis**		**Lymphatic Filariasis**		**Soil transmitted helminths**	
	**N**	**N (%)**	P value*	**N (%)**	P value*	**N (%)**	P value*	**N (%)**	P value*
**Age group (years)**									
14–22	153	54 (35∙3)		61(39∙9)		72(47∙1)		116(75∙8)	
23–30	210	48(22∙9)	**0∙04**	63(30∙0)	**0∙01**	99(47∙1)	0∙10	151(71∙9)	**0∙03**
>30	87	22(25∙3)		22(25∙3)		30(34∙5)		54(62∙1)	
**Education and literacy**									
Illiterate	82	25(30∙5)		30(36∙6)		44(53∙7)		70(85∙4)	
Primary	294	76(25∙9)	0∙97	99(33∙7)	0∙08	126(42∙9)	0∙13	206(70∙1)	**<0∙001**
Secondary	74	23(31∙1)		17(23∙0)		31(41∙9)		45(60∙8)	
**Household income**									
<1000–2999	9	2(22∙2)		4(44∙4)		2(22∙2)		6(66∙7)	
3000–4999	257	68(26∙5)	0∙45	97(37∙7)	**0∙003**	124(48∙3)	0∙35	192(74∙7)	0∙12
>5000	184	54(29∙4)		45(24∙5)		75(40∙8)		123(66∙9)	
**Ethnic group**									
Digo	395	109(27∙6)		131(33∙2)		176(44∙6)		283(71∙7)	
Duruma	21	5(23∙8)	0∙99	9(42∙9)	0∙11	11(52∙4)	0∙65	16(76∙2)	0∙40
Others	31	8(25∙8)		6(19∙4)		12(38∙7)		20(64∙5)	
**Parity**									
Primigravida	117	37(31∙6)		42(35∙9)		48(41∙0)		82(70∙1)	
Secundigravida	84	27(32∙1)	0∙10	29(34∙5)	0∙25	38(45∙2)	0∙37	59(70∙2)	0∙64
Multigravida	249	60(24∙1)		75(30∙1)		115(46∙2)		180(72∙3)	

* P-value for a significant difference in the infection frequency among the three groups for each characteristic. Significant values (<0.05) are indicated in **bold**

### Maternal infections

**Figure 2:**
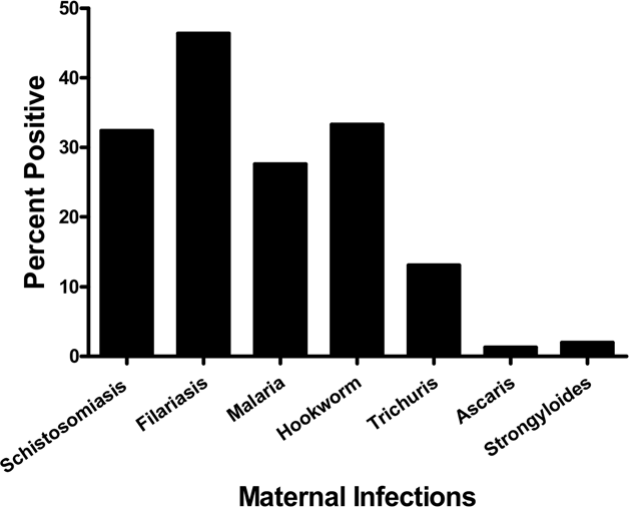
Prevalence of malaria and helminth infections in pregnant mothers.

Overall, 78·9% of the mothers were infected with one or more of the following infections; *P. falciparum* malaria, lymphatic filariasis (LF), schistosomiasis and/or intestinal helminths (Fig. 2). Prevalence of *Strongyloides* and *Ascaris* was <2%. Polyparasitism proved common in our cohort: a single infection was detected in 29·6%, two infections in 27·6%, three infections in 15·6%, four infections in 4·2% and five infections in 1·3% of women.

### Associations between maternal malaria and/or helminth infection and anti-vaccine IgG levels in children

**Figure 3:**
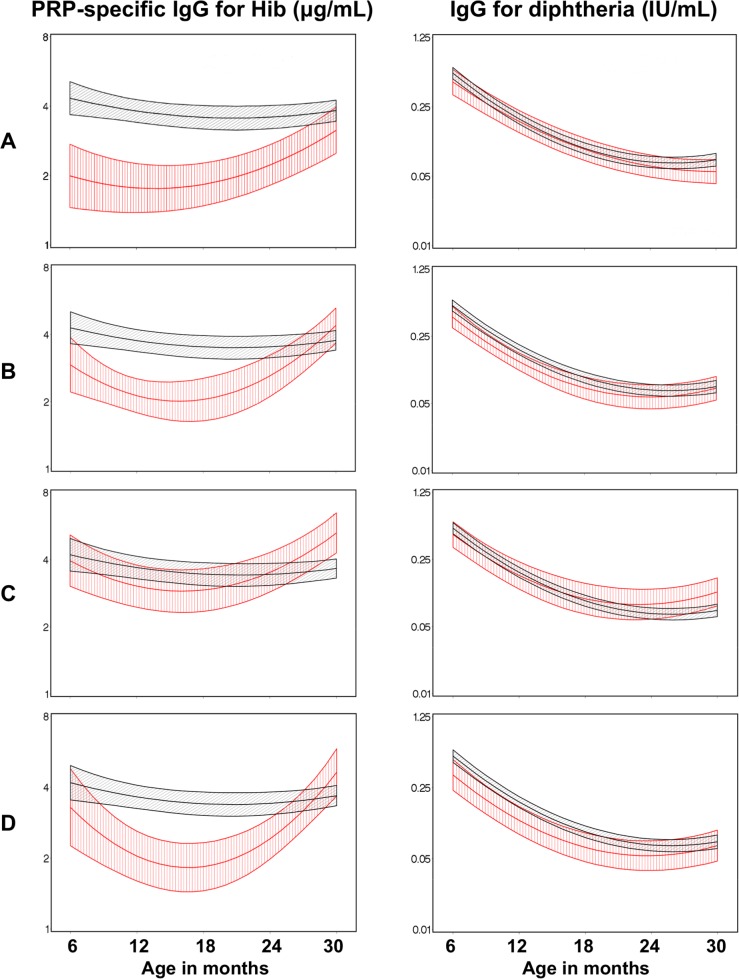
Effects of maternal parasitic infections on post-vaccination IgG to *Haemophilus influenzae* b and to diphtheria. Shown are adjusted mean anti-Hib polyribitol phosphate (PRP) (left panels) and anti-diphtheria toxoid antibody levels (right panels) among infants born to mothers who had (red line), or did not have (black line) the indicated prenatal maternal infection; Row A: maternal malaria; Row B: maternal filariasis; Row C: maternal schistosomiasis; Row D: maternal hookworm. Estimates were obtained using a generalized non-linear mixed model that included fixed effects for maternal age, parity, occupation, education and child infections, a quadratic fixed effect for age of infant, and a random intercept. Hash lines indicate the standard error of the mean values.

To determine whether the presence of individual infection during pregnancy was associated with impaired IgG responses to Hib, DT, Hep B, and TT in infancy and early childhood, children were stratified into groups whose mothers were independently malaria-, schistosomiasis-, LF-, or hookworm-infected, or not infected during pregnancy; other STH, such as *Trichuris*, *Strongyloides* were detected in <2% and *Ascaris*, was detected in <11% of the mothers and therefore were not considered further in the analysis. The presence of pre-natal maternal malaria, LF, and hookworm (Fig. 3, rows A, B, and D respectively) was associated with significantly lower post-vaccination levels of Hib-specific IgG (for malaria, P = 0•031, 0•005, 0•007, 043 at 6, 12, 18, 24 months of age; for LF, P = 0•007, 0•03 at 12, 18 months of age; and for hookworm, P = 0•034, 0•019 at 12, 18 months of age, respectively, controlling for maternal age, parity, education, income, and childhood parasitic infections). Maternal schistosomiasis alone (Fig. 3, row C) had no effect on vaccine-specific IgG levels. Malaria, LF, schistosomiasis and hookworm had no effect on DT, (Fig. 3), Hep B and TT (see Supporting Information, S1 Fig.).

**Figure 4:**
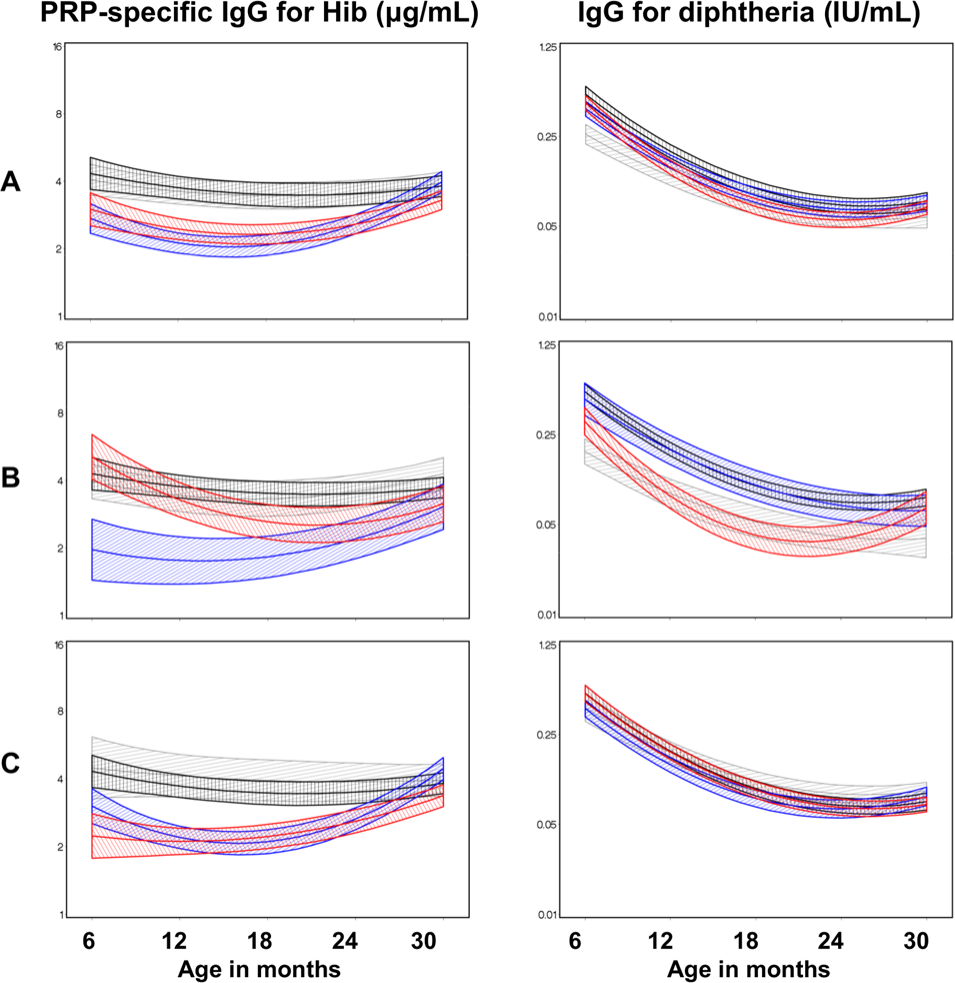
Response to vaccines in infants of mothers with multiple infections. Adjusted mean anti-Hib polyribitol phosphate (PRP) (left panels) and anti-diphtheria toxoid antibody levels (right panels) from 6 to 30 months of age, according to the multiplicity of the mother’s pre-natal infections. Row A: No maternal infection (black line), one maternal parasitic infection (blue line), two maternal parasitic infections (red line), three or more maternal parasitic infections (grey line). Row B. No maternal infection (black line), maternal malaria infection alone (blue line), maternal malaria infection and one helminth (red line), maternal malaria and two or more helminth infections (grey line). Row C: No maternal infection (black line), single maternal helminth and no malaria (blue line), two helminth infections and no malaria (red line), and three or more helminth infections and no malaria (grey line).

The presence and the multiplicity of prenatal parasitic infections were significantly associated with reduced IgG responses to vaccine antigens during infancy following pentavalent vaccination given at 6, 10, and 14 weeks of age. Compared to no prenatal maternal infection, the presence of single or double maternal infections during pregnancy resulted in significantly lower anti-Hib PRP-specific IgG levels in infants and young children to 2 years of age (Fig. 4 row A); (P = 0·04, <0·0001, 0·001, 0·04 with one infection for 6, 12, 18, and 24 month time points, and P = 0·008, 0·01, 0·04 with two infections at 12, 18, and 24 months of age, respectively). Offspring of mothers with 3 or more parasitic infections did not have reduced anti-Hib PRP-IgG (Fig. 4, row A). Prenatal parasitic infections had little impact on DT-specific IgG levels in infants of mothers with one or two infections, but there was an effect in infants of mothers with three or more infections at 6 and 12 months of age, (Fig. 4, row A, P = 0·002 and 0·03, respectively).

To better understand the interaction of different parasitic infections in mothers on vaccine responses in their offspring, we examined the response to vaccine antigens in infants of uninfected mothers versus those infected with: i) malaria alone, ii) malaria plus one helminth and iii) malaria plus two or more helminths. As shown in Fig. 4, row B, response to Hib was significantly lower in infants of mothers with single malaria infection (P = 0·027, 0•005, 0•007, and 0•046 at 6, 12, 18, and 24 months of age); whereas response to DT was lower in infants of mothers with malaria plus one helminth (P = 0•001, 0•003, and 0•024 at 12, 18, and 24 months) and malaria plus two or more helminths (P = 0•0001, 0•005, 0•037, and 0•022 at 6, 12, 18 and 24 months). We also examined the response to vaccines in infants of uninfected mothers versus those infected with: i) one helminth and no malaria, ii) two helminths and no malaria, and iii) three or more helminths and no malaria (Fig. 4, row C). The Hib-specific IgG levels were lower in infants of mothers with single helminth and no malaria (P = 0•001 and 0•003 at 12 and 18 months) and two helminths and no malaria (P = 0•018, 0•002, and 0•011 at 6, 12, and 18 months). In contrast, there was no significant difference in TT or Hep B-specific post-vaccination IgG levels in infants of mothers with multiple infections as compared to mothers with no infection (S2 Fig.).

### Effects of sensitization and tolerization on response to vaccine antigens

**Figure 5:**
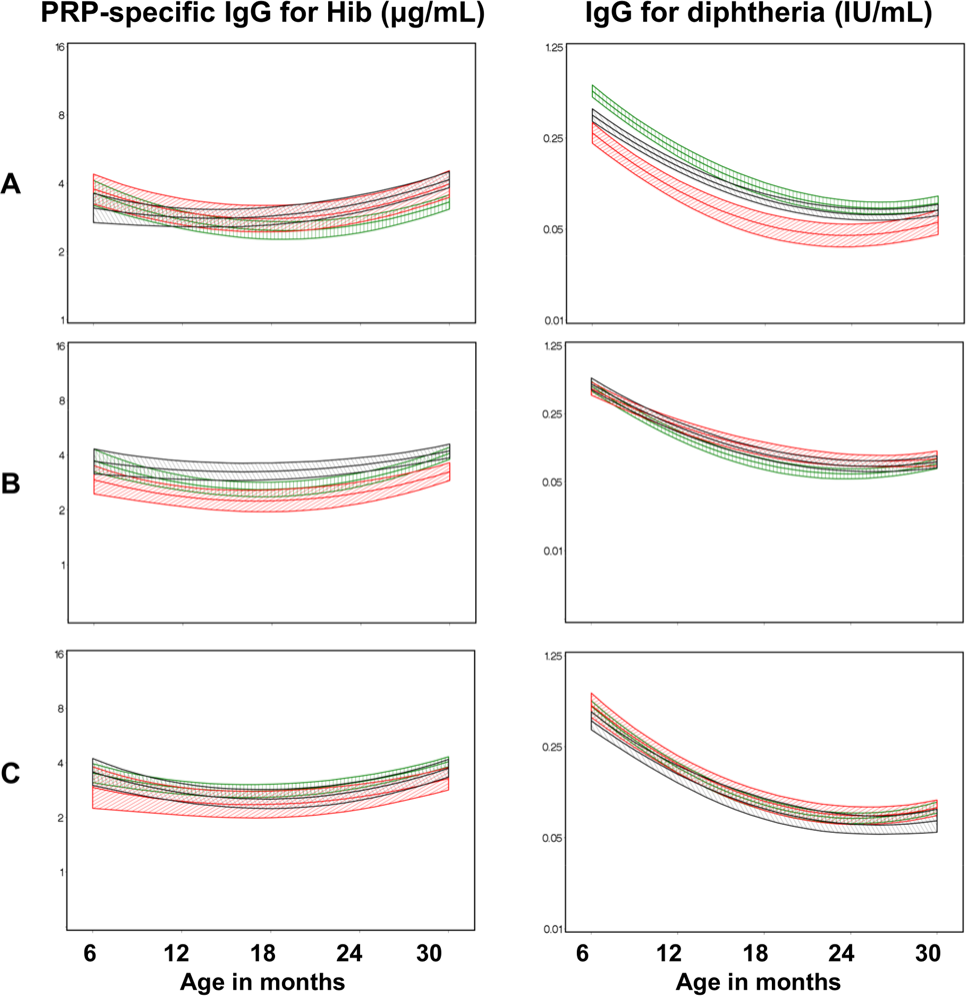
Effects of malaria, LF, and *S. haematobium* sensitization and tolerization on response to vaccine antigens. Adjusted mean antibody responses to Hib polyribitol phosphate (PRP) and diphtheria toxoid antigens among infants who were unexposed to parasite infections *in utero*(black line), exposed-sensitized (green line), or exposed-tolerized (red line), based on cord blood anti-parasite reactivity at birth; Row A: maternal malaria; Row B: maternal filariasis; Row C: maternal schistosomiasis.

Children were classified as either sensitized, tolerized, or unexposed to the individual parasite pathogens studied, as described in detail in the methods. With respect to Hib vaccine, classification of children by cord blood response to prenatal malaria had no effect on anti-Hib Ab levels (Fig. 5, row A), however offspring who developed a tolerogenic response to filarial antigens had significantly reduced anti-Hib responses at 12, 18, and 24 months of age (P = 0·052, 0·033, and 0·035, respectively, Fig. 5, row B). There was no impact of cord blood response to prenatal schistosome exposure on anti-Hib IgG levels (Fig. 5, row C). By contrast immunophenotype acquired by prenatal exposure to malaria antigens had significant effects of development anti-DT IgG levels following vaccination. Children classified as malaria-tolerant showed significantly reduced DT-specific IgG levels at 12 and 18 months of age, (P = 0·032 and 0·048, respectively) when compared to unexposed infants. There were *increased* anti-DT IgG levels in malaria-sensitized infants at 6 and 12 months (P = 0·006 and 0·04 respectively, Fig. 5, row A).

To determine if the sensitization in this group of infants was to malaria antigens alone or a T cell response to malaria plus other antigens, we further analyzed the sensitized group looking at the Th1/Th2-type recall responses to malaria alone, malaria+SWAP and malaria+BMA. There was no difference in anti-DT IgG in infants sensitized to malaria alone, but infants sensitized to malaria+SWAP showed a relative *increase* in DT-specific IgG levels. Children classified as tolerized to LF (based on Og4C3 or BMA-specific IgG4 positivity of their mothers) had levels of anti-Hib IgG that were significantly reduced at 12, 18, and 24 months of age (P = 0·052, 0·033, and 0·035, respectively, Fig. 5, row B). There were no significant differences in the levels of IgG response to Hib, DT, Hep B, and TT (Fig. 5, as well as S3 Fig.) among *Schistosoma-*tolerized or-sensitized infants as compared to the responses of those who were unexposed.

## Discussion

This study shows that there is an impaired ability to develop IgG antibody responses to key protective antigens of Hib and diphtheria among infants of mothers infected with malaria and/or helminths during pregnancy, as compared to infants of uninfected mothers. This association with maternal infection was most pronounced for anti-Hib antibody responses, with some evident effects on diphtheria antibody response, but no measurable effect on acquisition of antibodies to TT and hepatitis B. Independently, antenatal malaria, LF, and hookworm each lowered the IgG response to Hib, but schistosomiasis had no effect. Most pregnant women were infected with more than one of these parasitic infections, yet having multiple infections did not increase the observed impaired levels of antibody responses, suggesting that single species-specific mechanisms may be responsible for this vaccine-response impairment.

To examine the possibility that maternal parasitic infections represented only a proxy for other factors that could impact children’s responses to vaccination, we adjusted for the effects of maternal age, parity, occupation, income, and acquisition of childhood parasitic infections. None of these co-factors had a significant effect on the strength of association of maternal infection with antibody responses, indicating there could indeed be a causal association between maternal parasitic infections and impaired vaccine responses. We hypothesized that the effects of prenatal maternal malaria or helminth infections could be based on *in utero* modification of fetal immune responses by transplacental exposure of the fetus to parasite antigens [29, 31, 37–39]. Therefore, the vaccination outcomes among offspring of infected women were further analyzed after classification of the infant study subjects into sensitized and putatively tolerant groups, based on their cord blood responses to identified maternal infections. Tolerance is likely to contribute to the long-term persistence of many intravascular parasitic infections and this phenomenon is associated with impaired or altered fetal immune response by generation of regulatory T cells [40–42]. Not all *in utero* exposure to maternal helminth infections results in tolerance. Instead, for some newborns, prenatal parasite exposure results in a constant state of anti-parasite immune activation that is characterized by a Th2-dominant cytokine profiles, *i.e.*, high IgE levels, eosinophilia, and generation of regulatory T cells. Such an immune profile could have an adverse impact on the efficacy of vaccines by limiting Th1-type pathways of immune response to vaccination. By altering the immunologic balance between Th1-type and Th2-type pathways and generation of regulatory T cells, chronic parasitic infections appear to alter the immunologic milieu and could also likely impair or suppress the ‘‘normal’’ responses to vaccines that have been described in parasite-free, developed countries. Our data suggest that the malaria-tolerized group was less likely to respond well to DT, and that the filarial-tolerized group was less likely to respond well to Hib, when compared to unexposed children. By contrast, the schistosomiasis-tolerized group showed no evident effect on the vaccination responses we studied.

Interestingly, maternal helminth infections, in the absence of malaria infection, had no effect on antibody responses to DT, TT and hepatitis B, whereas maternal malaria infection with one or more helminth infection resulted in a significant drop in antibody responses to DT, and not to TT or hepatitis B. This impaired response to DT was most pronounced in children who were classified as immune tolerant. The differential impact of maternal infections on immune responses to various vaccines may arise from the type of vaccine and breadth of antibody responses. In contrast to DT, TT, and hepatitis B, Hib is a polysaccharide vaccine conjugated to TT and the antibody responses is directed to polyribosylribitol phosphate, an oligosaccharide with two protective epitopes [43], and thus a very restricted epitope repertoire. Importantly, a low antibody to PRP correlates with impaired vaccine efficacy (<0.15 ug/ml). Even though most children with impaired PRP antibody response had anti-PRP above the protective threshold, lower initial steady-state anti-PRP IgG antibody response could lead to a reduced long-term immunological memory [44]. DT and TT are complex antigen mixtures containing many bacterial pathogen-associated molecular patterns (PAMPs) that may elicit a more pro-inflammatory cytokines that may circumvent any antenatal acquired immunoregulatory response. In addition, antibody responses measured to these crude antigenic mixtures may mask a tolerogenic effect to certain epitopes. An epitope-specific tolerogenic responses has been observed to the malaria surface protein 1 (MSP1) in offspring of mice infected with malaria [45].

Results from the current study support the hypothesis that antenatal single and multiple parasitic infections can imprint effects on fetal immunity, and thereby affect certain infant vaccine responses in the first months after birth. Both animal and human studies indicate that parasitic infections can have an effect on long-term responses to vaccination. Chronic *concurrent* parasitic infections have proven to have harmful immune effects including decreased tetanus, Hib, and typhoid vaccine responses in the presence of active malaria infection in humans [14, 46]. By contrast, studies have shown no effect of maternal infection with *T. cruzi*or congenital Chagas disease on responses to BCG, hepatitis B, diphtheria, or tetanus vaccination in the neonatal period [47]. A recent randomized, placebo-controlled trial did not find an effect for antenatal anti-helminthic treatment (of low intensity infections) on infant responses to tetanus, BCG, or measles vaccinations, although a small effect was noted for tetanus response when the analysis was focused only on the subgroup of mothers known to be infected [48]. In our study, we observed lower IgG responses to Hib and DT, but not to tetanus. This may be because the pregnant mothers were immunized with tetanus vaccine according to Ministry of Health guidelines, and the maternal antibodies during pregnancy and later infant responses were not affected by antenatal malaria or helminth infection. In past studies, active schistosomiasis in humans has been shown to have a negative impact on responses to tetanus and BCG vaccination [14]. However, we did not observe an effect of maternal schistosomiasis alone on tetanus, Hep B, DT, or Hib responses.

There were strengths and limitations in the present study. In our study, the detection of malaria, schistosomiasis and LF infections utilized very sensitive and specific experimental detection assays to classify infection status. By avoiding the sole use of stool and urine examinations, which are less sensitive and could miss low level infections, our results provide realistic estimates of STH and *S. haematobium* infections. Conversely the reliance on serological assay to detect most of the *Schistosoma* infections, in particular, may have overestimated ongoing infections or detected such light infections that they were unlikely to have significantly exposed the fetus to parasite antigens. Our long-term, prospective follow-up of a large cohort of mother-infant pairs allowed for better definition of the time-related pattern of vaccine response at the individual level. The outcomes of the present study are just based on the antibody responses to Hib, DT, TT, and Hep B vaccine antigens. Other vaccines are given in the study site, and analysis of these will be included in future studies. The children were vaccinated for Hib, DT, TT, and Hep B at 6, 10 and 14 weeks, but vaccine response was not measured until 6 months of age, so the children’s immediate responses to vaccination are not known. It is possible that children who were exposed to natural infection during the observation intervals may have had varied vaccine responses and that we may have missed differences between our groups in their post-primary and post-secondary responses. Similarly, an in-depth assessment of maternal and child nutrition was not done in this study, and aspects of macro- and micro-nutrient intake may have had confounding effects on infection and response to vaccines [49]. Finally, our prenatal infection data include only maternal malaria and helminth infections at the first antenatal visit and at the time of delivery; mothers may have been exposed to these and other infections during pregnancy without detection by the study’s testing. Despite these limitations, we feel that this study offers an accurate documentation of the effect of antenatal parasitic infection on IgG antibody response to vaccine-preventable disease antigens.

Given the complexity of parasitic impact on fetal immunity, our results suggest that antenatal malaria, LF, schistosomiasis, and hookworm variously affect the levels of post-vaccination protection against Hib and diphtheria among infants between 6–30 months of age. While the observed lower levels of antibody response may not obviate the individual protective effect of vaccination, these lower levels of immune response could contribute to persistent pathogen carriage, leading to continuing disease transmission among affected communities. Many of the effects we found were parasite-specific and vaccine-dependent, suggesting that parasitic effects can differ depending on the type and the intensity of infections. To explore the duration of the observed effects, we are in the process of re-examining our study cohort, now aged 5–8 years old, and testing their immune responses to new vaccine antigens. Overall, the present findings reinforce the importance of control and prevention of parasitic infections in pregnant women. They also suggest that if vaccine efficacy is to be ensured in disease-endemic developing countries, eradication of chronic helminthic infections may be imperative to the overall success of global vaccination efforts.

## Supporting Information

S1 Fig Effects of maternal parasitic infections on post-vaccination IgG to Hep B and TT. Adjusted mean anti-Hepatitis B and anti-tetanus toxoid antibody levels in infants of mothers with (red line) or without (black line) the selected prenatal maternal infection; Row A: maternal malaria; Row B: maternal filariasis; Row C: maternal schistosomiasis; Row D: maternal hookworm.
(DOCX)

S2 Fig Response to vaccines in infants of mothers with multiple infections. Adjusted mean anti-Hepatitis B and anti-tetanus toxoid antibody levels from 6 to 30 months of age, according to the multiplicity of the mother’s pre-natal infections. Row A: No maternal infection (black line), one maternal parasitic infection (blue line), two maternal parasitic infections (red line), three or more maternal parasitic infections (grey line). Row B. No maternal infection (black line), maternal malaria infection alone (blue line), maternal malaria infection and one helminth (red line), maternal malaria and two or more helminth infections (grey line). Row C: No maternal infection (black line), single maternal helminth and no malaria (blue line), two helminth infections and no malaria (red line), and three or more helminth infections and no malaria (grey line).
(DOCX)

S3 Fig Effects of malaria, LF, and *S. haematobium* sensitization and tolerization on response to vaccine antigens. Adjusted mean antibody responses to Hepatitis B and tetanus toxoid antigens in infants who were unexposed to parasite infections *in utero* (black line), exposed-sensitized (green line), or exposed-tolerized (red line), based on cord blood anti-parasite reactivity at birth; Row A: maternal malaria; Row B: maternal filariasis; Row C: maternal schistosomiasis.
(DOCX)
