# Supplementary figures and images for: Effect of Antenatal Parasitic Infections on Anti-vaccine IgG Levels in Children: A Prospective Birth Cohort Study in Kenya
Source: PLoS Negl Trop Dis. 2015 Jan 15;9(1):e0003466. doi: 10.1371/journal.pntd.0003466 (PMC4295886; doi:10.1371/journal.pntd.0003466)

**Figure S2**

**
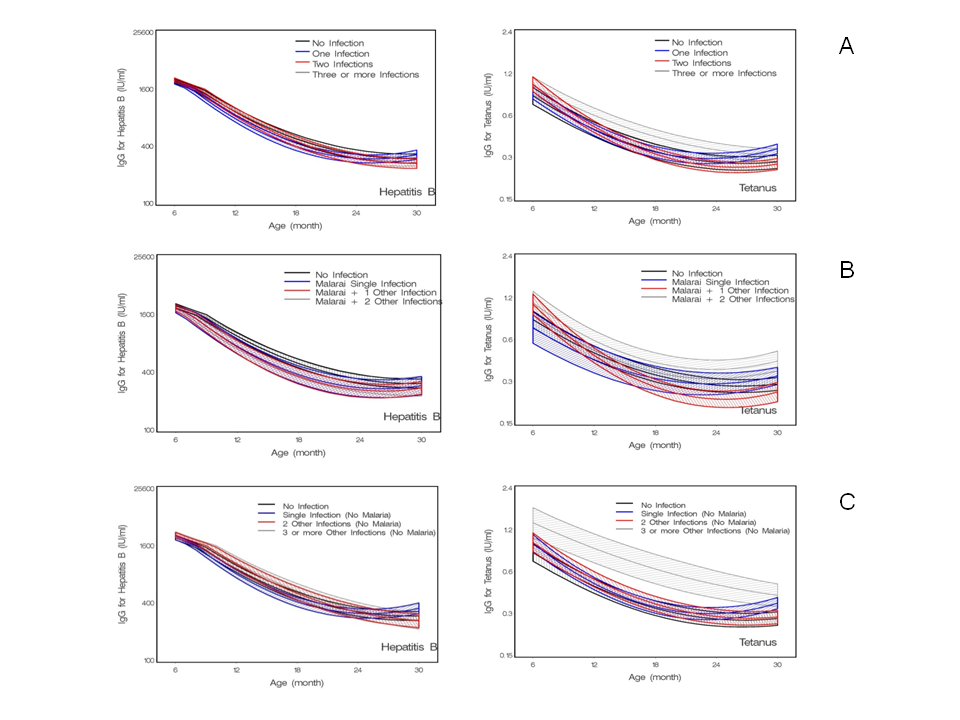
**

Supplement: S2 Fig — Adjusted mean anti-Hepatitis B and anti-tetanus toxoid antibody levels from 6 to 30 months of age, according to the multiplicity of the mother’s pre-natal infections. Row A: No maternal infection (black line), one maternal parasitic infection (blue line), two maternal parasitic infections (red line), three or more maternal parasitic infections (grey line). Row B. No maternal infection (black line), maternal malaria infection alone (blue line), maternal malaria infection and one helminth (red line), maternal malaria and two or more helminth infections (grey line). Row C: No maternal infection (black line), single maternal helminth and no malaria (blue line), two helminth infections and no malaria (red line), and three or more helminth infections and no malaria (grey line). (DOCX) [file pntd.0003466.s002.docx]

**Figure S3**


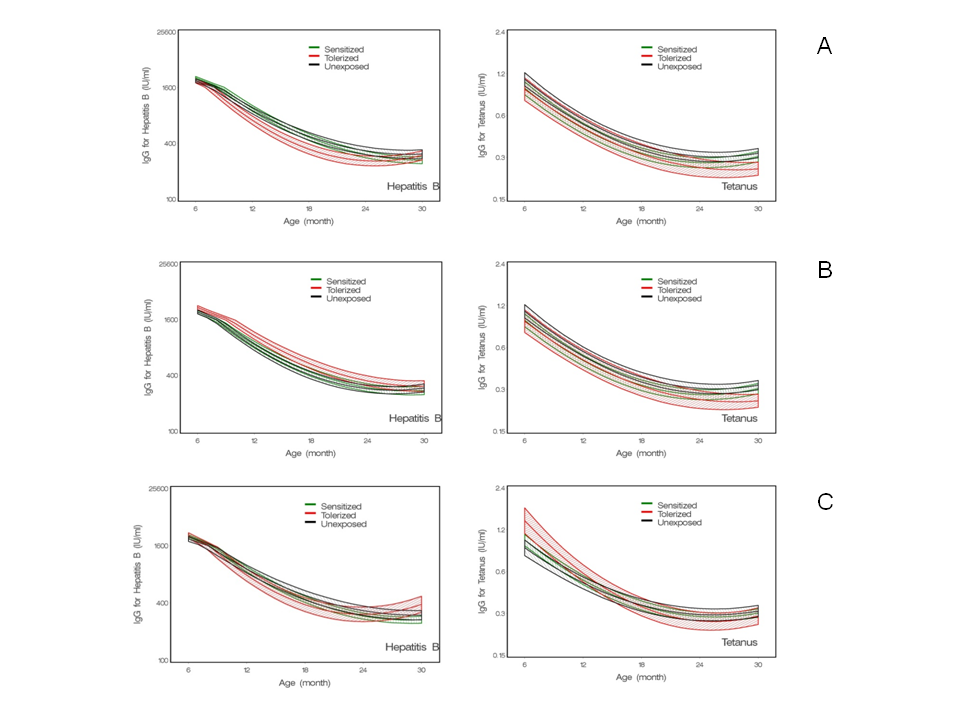

Supplement: S3 Fig — Adjusted mean antibody responses to Hepatitis B and tetanus toxoid antigens in infants who were unexposed to parasite infections in utero (black line), exposed-sensitized (green line), or exposed-tolerized (red line), based on cord blood anti-parasite reactivity at birth; Row A: maternal malaria; Row B: maternal filariasis; Row C: maternal schistosomiasis. (DOCX) [file pntd.0003466.s003.docx]
